# Anxiety and depressive symptoms and health-related quality of life in girls with central precocious puberty versus isolated premature thelarche: a comparative cross-sectional study

**DOI:** 10.3389/fpsyt.2026.1971843

**Published:** 2026-10-16

**Authors:** Burak Kamış, Semine Özdemir Dilek, Gürkan Tarçın

**Affiliations:** 1 Department of Child and Adolescent Psychiatry, Adana City Training and Research Hospital, Adana Faculty of Medicine, University of Health Sciences, Adana, Türkiye; 2 Department of Pediatric Endocrinology, Adana City Training and Research Hospital, Adana Faculty of Medicine, University of Health Sciences, Adana, Türkiye; 3 Department of Pediatric Endocrinology, Faculty of Medicine, Kocaeli University, Kocaeli, Türkiye

**Keywords:** anxiety, central precocious puberty, children, depressive symptoms, health-related quality of life, isolated premature thelarche

## Abstract

**Introduction:**

Previous studies have examined psychological symptoms and health-related quality of life (HRQoL) in girls with central precocious puberty (CPP) and isolated premature thelarche (IPT), but findings from direct comparisons have been inconsistent. We aimed to compare anxiety and depressive symptoms and HRQoL between girls with CPP and IPT and to examine associations between psychological symptom burden and HRQoL.

**Methods:**

This comparative cross-sectional study included 50 girls (mean age, 8.33 ± 0.23 years), including 18 with idiopathic CPP and 32 with IPT. Breast development had first been recognized before 8 years of age in all participants. Psychological questionnaires were administered on the same day as the endocrinological evaluation. Anxiety and depressive symptoms were assessed using SCARED, RCADS-CV, and CDI, and self-reported HRQoL was assessed using PedsQL 4.0. Between-group comparisons incorporated Benjamini–Hochberg false-discovery-rate correction. Spearman correlations and exploratory hierarchical linear regression were performed.

**Results:**

No between-group differences in anxiety, depressive symptoms, or HRQoL remained statistically significant after FDR correction. Clinically elevated SCARED scores occurred in 38.9% of girls with CPP and 53.1% with IPT (p=0.333), while CDI scores ≥19 occurred in 11.1% and 18.8%, respectively (p=0.694). In the combined sample, psychological symptom scores were inversely correlated with PedsQL total scores (Spearman’s r_s_=−0.568 to −0.720; all p<0.001). In exploratory regression adjusted for endocrine phenotype, BMI SDS, and symptom duration, SCARED (β=−0.382, p=0.003) and CDI (β=−0.481, p<0.001) scores were associated with lower PedsQL total scores.

**Discussion:**

Higher anxiety and depressive symptom scores were associated with lower HRQoL in this clinical sample. The absence of statistically significant CPP–IPT differences does not establish equivalence or exclude a contribution of endocrine factors. Larger longitudinal studies are needed to confirm these associations.

## Introduction

Central precocious puberty (CPP) is characterized by the onset of progressive pubertal development before the age of 8 years in girls resulting from premature activation of the hypothalamic–pituitary–gonadal (HPG) axis (1). In contrast, isolated premature thelarche (IPT) refers to isolated breast development beginning before the age of 8 years without other progressive pubertal signs and typically without sustained central HPG-axis activation (2).

Psychological and behavioral outcomes in precocious puberty have been investigated, but findings remain heterogeneous (3). Çoban et al. (4) reported greater peer victimization among girls with CPP than among healthy controls, without statistically significant differences in depressive symptoms or health-related quality of life (HRQoL). Kardaş et al. (5) found greater anxiety and poorer HRQoL among girls referred for concerns about pubertal development; child outcomes were also associated with maternal anxiety and pubertal stage. Neuroimaging research has identified associations between central pubertal activation, prefrontal cortical morphology, and behavioral measures, although these observations do not establish that hormonal activation causes psychiatric symptoms (6).

Other clinical investigations have reported increased psychiatric morbidity in girls with precocious puberty (7), while population-based cohorts have identified elevated risks of psychiatric disorders during follow-up (8, 9). A recent clinical study also reported more anxiety disorders and difficulties in selected psychosocial domains among girls with newly diagnosed idiopathic CPP than among healthy controls (10). These studies support the clinical relevance of psychological assessment but do not establish whether contemporaneous symptom profiles differ between girls with CPP and those with IPT.

Direct comparisons of CPP and premature thelarche have also produced inconsistent findings. Yılmazer et al. (11) found no statistically significant overall differences in physical, psychosocial, or total Pediatric Quality of Life Inventory (PedsQL) scores among children with CPP, premature thelarche, and healthy controls. In contrast, Donbaloglu and Bostan (12) reported greater anxiety–depression symptom burden and poorer HRQoL in girls with premature thelarche than in those with idiopathic CPP. In a separate cross-sectional study, Köprülü and Yazkan Akgül (13) found no statistically significant differences in anxiety–depression scores or HRQoL among children with newly diagnosed CPP, children receiving treatment for CPP, and healthy controls. Taken together, these findings indicate an existing but heterogeneous evidence base and warrant further direct CPP–IPT comparisons to assess the consistency of psychological and HRQoL differences across clinical samples.

Accordingly, the present study compared anxiety and depressive symptom severity and multidimensional health-related quality of life between girls with CPP and those with IPT. We also examined whether anxiety and depressive symptom scores were associated with HRQoL in the combined sample. This cross-sectional comparison was intended to characterize clinical associations, not to isolate endocrine effects from unmeasured psychosocial influences or to estimate excess psychiatric morbidity relative to healthy peers.

## Materials and methods

### Study design and setting

This comparative cross-sectional study was conducted jointly by the Departments of Pediatric Endocrinology and Child and Adolescent Psychiatry at the University of Health Sciences, Adana Faculty of Medicine, Adana City Training and Research Hospital, Adana, Türkiye. The study protocol was approved by the Adana City Training and Research Hospital Clinical Research Ethics Committee on 17 August 2023 (meeting no. 133, decision no. 2776). Participant recruitment and study-specific data collection commenced only after ethics approval had been obtained and continued through November 2023.

Girls presenting to the pediatric endocrinology outpatient clinic with breast development beginning before 8 years of age were consecutively evaluated for eligibility. The study compared two clinically defined endocrine phenotypes: idiopathic central precocious puberty (CPP) and isolated premature thelarche (IPT). No healthy-control group was included. The final analytical sample comprised 50 girls, including 18 with CPP and 32 with IPT.

Written informed consent was obtained from parents or legal guardians, and age-appropriate written assent was obtained from the children where applicable. The study was conducted in accordance with the Declaration of Helsinki, applicable local legislation, and institutional requirements.

### Participants and eligibility criteria

Eligibility required parent-reported first recognition of breast development before 8 years of age, an age younger than 9 years at study assessment, and an endocrinological diagnosis of idiopathic CPP or IPT. The upper assessment-age limit allowed for the interval between symptom recognition and referral for pediatric endocrinological evaluation while restricting the chronological age range of the study population.

Age at first recognition of breast development was obtained from the parental history documented in the individual clinical records and was distinguished from age at study assessment. Symptom duration was recorded in months and represented the interval between the parent-reported first recognition of breast development and the study assessment.

Exclusion criteria included an identifiable organic cause of precocious puberty; chronic systemic, neurological, or psychiatric disease; genetic or syndromic disorders; and inability to complete the study assessments because of communication limitations. Participants with conditions potentially affecting pubertal development, including congenital adrenal hyperplasia, hypothyroidism, ovarian or adrenal pathology, or exogenous estrogen exposure, were also excluded. Additional exclusion criteria were previous or ongoing treatment with a gonadotropin-releasing hormone (GnRH) agonist, use of psychotropic medication, and incomplete clinical, laboratory, or GnRH stimulation-test data required for diagnostic classification. For participants with CPP, missing or incomplete brain and pituitary magnetic resonance imaging (MRI) data that precluded evaluation for an organic central nervous system etiology also constituted an exclusion criterion.

### Clinical and anthropometric assessment

All participants underwent a standardized pediatric endocrinological evaluation. Pubertal development was assessed according to Marshall and Tanner (14), and thelarche was defined as Tanner breast stage II (B2) or higher. Breast development was confirmed by palpation to distinguish true glandular breast tissue from lipomastia, particularly in girls with overweight or obesity.

Height and weight were measured using standardized techniques, and body mass index (BMI) was calculated as weight in kilograms divided by height in meters squared. Height, weight, and BMI standard deviation scores (SDS) were calculated using age- and sex-specific Turkish reference data reported by Neyzi et al. (15), with calculations performed using the ÇEDD Çözüm/TPEDS Metrics (Child Metrics) calculator described by Demir et al. (16).

For descriptive analyses, weight status was classified using BMI-SDS thresholds corresponding to conventional percentile categories: underweight (<5th percentile; SDS <−1.645), normal weight (5th to <85th percentile; SDS −1.645 to <1.036), overweight (85th to <95th percentile; SDS 1.036 to <1.645), and obesity (≥95th percentile; SDS ≥1.645). For categorical comparisons, overweight and obesity were combined as BMI ≥85th percentile and compared with underweight or normal weight.

### Endocrinological evaluation

The diagnostic evaluation included physical examination, measurement of basal luteinizing hormone (LH), follicle-stimulating hormone (FSH), and estradiol concentrations, bone-age assessment, pelvic ultrasonography, and a GnRH stimulation test.

Serum LH, FSH, and estradiol concentrations were measured using a chemiluminescent immunoassay on the UniCel DxI 800 Access Immunoassay System (Beckman Coulter, Brea, CA, USA).

Bone age was determined from a radiograph of the left hand and wrist using the Greulich and Pyle atlas (17). Pelvic ultrasonography was performed as part of the differential diagnostic evaluation and was interpreted together with clinical, auxological, and biochemical findings.

### GnRH stimulation test and diagnostic classification

The GnRH stimulation test was used to assess hypothalamic–pituitary–gonadal (HPG) axis activation (18). During the study period, the test was performed in all participants as part of the standard diagnostic protocol at our center, irrespective of basal LH concentrations. The same stimulation protocol was applied in both groups to standardize the biochemical assessment supporting classification as CPP or IPT.

The test was conducted in the morning between 08:00 and 09:00 after an overnight fast. After collection of basal LH and FSH samples, GnRH (Ferring^®^, 0.1 mg/mL) was administered intravenously at a dose of 2.5 μg/kg, up to a maximum dose of 100 μg. Blood samples for LH and FSH measurements were obtained at 15, 30, 45, 60, and 90 minutes after GnRH administration.

CPP was diagnosed in girls with breast development before 8 years of age and biochemical evidence of central pubertal activation, defined as a stimulated peak LH concentration ≥5 IU/L during the GnRH stimulation test (19). All participants included in the CPP group fulfilled this peak LH criterion; no participant was included solely on the basis of an elevated LH/FSH ratio. Progressive breast development, accelerated linear growth, and advanced bone age were considered supportive findings but were not used as sole diagnostic criteria.

IPT was defined as isolated breast development before 8 years of age in the absence of other progressive pubertal signs and without biochemical evidence of central HPG-axis activation on the GnRH stimulation test. Clinical growth pattern, bone-age findings, and pelvic ultrasonographic findings were considered as part of the differential diagnostic assessment.

### Neuroimaging

All 18 girls in the CPP group underwent contrast-enhanced brain and pituitary MRI to investigate an underlying central nervous system etiology. CPP diagnosis itself prompted neuroimaging; no additional MRI-specific age or neurological selection criteria were applied. A comparable systematic imaging approach was reported by Helvacıoğlu et al. (19).

### Psychological assessment and timing

Psychological symptoms and health-related quality of life (HRQoL) were assessed using child self-report questionnaires. In both the CPP and IPT groups, all questionnaires were completed during the same study assessment visit, on the same day as the endocrinological diagnostic evaluation. Participants attended the Pediatric Endocrinology and Child and Adolescent Psychiatry outpatient clinics on that day. In participants with CPP, all psychological and HRQoL assessments were completed before the initiation of GnRH agonist treatment.

### Assessment of anxiety symptoms

Anxiety symptoms were assessed using the Turkish child self-report version of the Screen for Child Anxiety Related Emotional Disorders (SCARED; Turkish abbreviation, ÇATÖ) (20, 21). The SCARED is a 41-item instrument designed to assess anxiety symptoms in children and adolescents. Each item is scored on a three-point scale from 0 to 2, yielding a total score ranging from 0 to 82, with higher scores indicating greater anxiety symptom burden (20).

A total SCARED score of ≥25 was considered indicative of clinically elevated anxiety symptoms warranting further clinical assessment (20, 21). This cutoff was used as a screening threshold and was not interpreted as establishing an anxiety-disorder diagnosis.

Anxiety symptoms were additionally evaluated using the Turkish child version of the Revised Child Anxiety and Depression Scale–Child Version (RCADS-CV; Turkish abbreviation, ÇADÖ) (22, 23). The RCADS-CV comprises 47 self-report items rated on a four-point scale ranging from 0 (“never”) to 3 (“always”) and assesses anxiety and depressive symptom dimensions (22). Anxiety, depression, and total raw scores were calculated according to the scoring instructions, with higher scores indicating greater symptom burden. The Turkish version has demonstrated satisfactory psychometric properties in children and adolescents (23).

RCADS-CV raw scores were analyzed as dimensional measures of symptom severity rather than used to classify participants according to normative clinical thresholds. Turkish population-based T-score conversion tables appropriate to this cohort were not identified; therefore, externally normed T-scores were not used.

### Assessment of depressive symptoms

Depressive symptoms were assessed using the Turkish version of the Children’s Depression Inventory (CDI) (24, 25). The CDI is a 27-item child self-report instrument assessing cognitive, affective, and behavioral symptoms of depression. Each item contains three response options scored from 0 to 2 according to symptom severity, yielding a total score ranging from 0 to 54; higher scores indicate greater depressive symptom burden (24).

A CDI total score of ≥19 was considered indicative of clinically elevated depressive symptoms based on the Turkish validation study (25). This threshold was used for screening purposes and was not considered sufficient to establish a diagnosis of depressive disorder. The depression raw score derived from the RCADS-CV was additionally evaluated as a complementary dimensional measure of depressive symptom burden.

SCARED and the RCADS-CV anxiety score were used as complementary measures of anxiety symptoms, whereas CDI and the RCADS-CV depression score were used as complementary measures of depressive symptoms. SCARED and CDI provide domain-specific assessments, while RCADS-CV evaluates both symptom domains within a common multidimensional framework. These instruments were included to examine the consistency of findings across validated measures. No anxiety or depression measure was designated *a priori* as the primary outcome; comparisons involving these measures were therefore considered complementary and exploratory.

### Health-related quality-of-life assessment

Health-related quality of life was assessed using the Turkish child self-report version of the Pediatric Quality of Life Inventory™ Version 4.0 Generic Core Scales (PedsQL™ 4.0) (26, 27).

The instrument comprises 23 items across four domains: physical functioning (8 items), emotional functioning (5 items), social functioning (5 items), and school functioning (5 items). The validated Turkish child self-report version for children aged 8–12 years was used in all participants (27).

Items were reverse-scored and linearly transformed to a 0–100 scale, with higher scores indicating better self-reported HRQoL. The Psychosocial Health Summary Score was calculated as the mean of the 15 items comprising the emotional, social, and school functioning domains, whereas the Total Scale Score was calculated as the mean of all 23 items (26).

In accordance with the PedsQL scoring instructions, a domain or summary score was not calculated when more than 50% of the corresponding items were missing. Before statistical analysis, physical, emotional, social, school, psychosocial, and total PedsQL scores were checked for valid ranges and internal arithmetic consistency with the standard scoring algorithm.

### Statistical analysis

Statistical analyses were performed using IBM SPSS Statistics version 26.0 (IBM Corp., Armonk, NY, USA). Before analysis, the dataset was checked for duplicate observations, implausible or out-of-range values, missing data, and inconsistencies in derived scale scores.

Continuous-variable distributions were assessed using the Shapiro–Wilk test and visual inspection. Normally distributed variables were summarized as mean ± standard deviation and compared using independent-samples t tests; non-normally distributed variables were summarized as median and interquartile range and compared using Mann–Whitney U tests. Categorical variables were presented as frequencies and percentages and compared using Pearson’s chi-square test or Fisher’s exact test, as appropriate.

The Benjamini–Hochberg false-discovery-rate (FDR) procedure was applied separately to two families of between-group comparisons: five psychological symptom scores—SCARED total, RCADS-CV anxiety, RCADS-CV depression, RCADS-CV total, and CDI total—and six PedsQL outcomes—physical, emotional, social, and school functioning, the Psychosocial Health Summary Score, and the Total Scale Score. Unadjusted p values and FDR-adjusted p values, reported as q values, were presented. Clinical and anthropometric comparisons, screening-threshold comparisons, correlation analyses, and exploratory regression analyses were outside these FDR families and were reported with unadjusted p values.

Spearman’s rank correlations were calculated between psychological symptom scores and PedsQL domain and summary scores in the combined sample. These analyses were exploratory.

An exploratory hierarchical multiple linear regression was performed with the PedsQL Total Scale Score as the dependent variable. Endocrine phenotype, BMI SDS, and duration of breast-development symptoms were entered in Step 1; SCARED and CDI total scores were added in Step 2. The change in R² was evaluated between steps. Model assumptions and potential influential observations were assessed using residual and normal probability plots, variance inflation factors, tolerance values, and Cook’s distance. Regression coefficients were interpreted as adjusted associations, not causal effects.

All tests were two-sided. Statistical significance was assessed at p<0.05 for unadjusted analyses and q<0.05 for FDR-adjusted comparisons, with unadjusted exploratory findings interpreted in the context of multiple testing.

## Results

### Participant and clinical characteristics

The final analytical sample comprised 50 girls, including 18 with central precocious puberty (CPP) and 32 with isolated premature thelarche (IPT). There were no missing observations for the principal anthropometric or psychological variables included in the analyses. Mean age at study assessment was 8.38 ± 0.26 years in the CPP group and 8.31 ± 0.20 years in the IPT group, with no statistically significant between-group difference (p = 0.322). The mean parent-reported age at first recognition of breast development was 7.99 ± 0.03 years in the CPP group and 7.86 ± 0.41 years in the IPT group (**Table 1**).

**Table 1:** Clinical and anthropometric characteristics of the study groups.

Variable	CPP (n=18)	IPT (n=32)	Test statistic	p
Age at study assessment, years, mean ± SD	8.38 ± 0.26	8.31 ± 0.20	Independent-samples t test	0.322
Parent-reported age at first recognition of breast development, years, mean ± SD	7.99 ± 0.03	7.86 ± 0.41	—	—
Duration since first recognition of breast development, months, median (IQR)	4.0 (3.0)	4.0 (6.0)	U = 284.0	0.935
Height SDS, mean ± SD	1.22 ± 1.00	0.91 ± 1.36	t=0.841	0.405
Weight SDS, mean ± SD	0.88 ± 1.05	1.05 ± 1.39	t=-0.460	0.647
BMI SDS, mean ± SD	0.44 ± 0.98	0.81 ± 1.12	t=-1.177	0.245
Overweight/obese, n (%)	5 (27.8)	14 (43.8)	χ²=1.247	0.264

Data are presented as mean ± SD, median (IQR), or n (%), as appropriate. Age at first recognition of breast development was obtained directly from parental history documented in the individual clinical records. Overweight/obesity was defined as BMI ≥85th percentile using the corresponding BMI-SDS threshold. CPP, central precocious puberty; IPT, isolated premature thelarche; SD, standard deviation; SDS, standard deviation score; BMI, body mass index; IQR, interquartile range; —, not applicable.

No statistically significant between-group differences were detected in height SDS, weight SDS, BMI SDS, duration of breast-development symptoms, or the proportion of participants with overweight or obesity (**Table 1**).

All 18 girls in the CPP group fulfilled the stimulated peak LH criterion of ≥5 IU/L and underwent contrast-enhanced brain and pituitary magnetic resonance imaging (MRI). No organic central nervous system etiology was identified in the final analytical CPP cohort.

### Anxiety and depressive symptoms

None of the five continuous psychological symptom-score comparisons was statistically significant after Benjamini–Hochberg false-discovery-rate (FDR) correction (**Table 2**).

**Table 2:** Anxiety and depressive symptom measures according to endocrine phenotype.

Measure	CPP (n=18)	IPT (n=32)	U/test	p	FDR q
SCARED total	17.50 (10.25)	25.00 (24.50)	222.0	0.181	0.453
RCADS-CV anxiety	28.5 (23.25)	26.5 (37.75)	272.5	0.754	0.754
RCADS-CV depression	3.0 (5.50)	5.5 (7.50)	188.5	0.043	0.215
RCADS-CV total	32.0 (31.50)	31.5 (46.25)	262.5	0.606	0.754
CDI total	6.5 (9.00)	9.5 (8.00)	241.5	0.346	0.577
SCARED ≥25, n (%)	7 (38.9)	17 (53.1)	χ²=0.935	0.333	—
CDI ≥19, n (%)	2 (11.1)	6 (18.8)	Fisher’s exact test	0.694	—

Continuous scale scores are presented as median (IQR). RCADS-CV values are raw scores, not standardized T-scores. Continuous scores were compared using the Mann–Whitney U test. SCARED and CDI thresholds represent clinically elevated screening scores and should not be interpreted as psychiatric diagnoses. The Benjamini–Hochberg procedure was applied to the five continuous psychological symptom-score comparisons shown in this table; FDR-adjusted p values are reported as q values. Screening-threshold comparisons were outside this FDR family and are reported with unadjusted p values. SCARED, Screen for Child Anxiety Related Emotional Disorders; RCADS-CV, Revised Child Anxiety and Depression Scale–Child Version; CDI, Children’s Depression Inventory; CPP, central precocious puberty; IPT, isolated premature thelarche; FDR, false discovery rate; IQR, interquartile range; —, FDR adjustment not applied.

Clinically elevated SCARED screening scores (≥25) were observed in 7/18 girls with CPP (38.9%) and 17/32 girls with IPT (53.1%) (p = 0.333). Clinically elevated CDI screening scores (≥19) occurred in 2/18 girls with CPP (11.1%) and 6/32 girls with IPT (18.8%) (Fisher’s exact p = 0.694). Neither screening-threshold comparison was statistically significant (**Table 2**).

### Health-related quality of life

No statistically significant between-group differences were detected in any PedsQL domain, the Psychosocial Health Summary Score, or the Total Scale Score, either before or after FDR correction (**Table 3**).

**Table 3:** PedsQL scores according to endocrine phenotype.

PedsQL domain	CPP median (IQR)	IPT median (IQR)	U	p	FDR q
Physical functioning	78.00 (19.58)	78.05 (22.38)	263.5	0.620	0.620
Emotional functioning	87.50 (33.75)	72.50 (38.75)	236.5	0.295	0.443
Social functioning	95.00 (16.25)	90.00 (23.75)	247.5	0.404	0.485
School functioning	87.50 (32.50)	77.50 (28.75)	229.5	0.235	0.443
Psychosocial Health Summary Score	90.00 (22.92)	80.00 (23.39)	234.5	0.279	0.443
Total Scale Score	86.95 (24.18)	78.33 (25.12)	235.5	0.288	0.443

Higher PedsQL scores indicate better self-reported health-related quality of life. Scores were compared using the Mann–Whitney U test. The Benjamini–Hochberg procedure was applied to the six PedsQL comparisons shown in this table; FDR-adjusted p values are reported as q values. The values in parentheses represent IQR widths. PedsQL, Pediatric Quality of Life Inventory; CPP, central precocious puberty; IPT, isolated premature thelarche; FDR, false discovery rate; IQR, interquartile range.

### Exploratory associations between anxiety and depressive symptom burden and HRQoL

In the combined sample (n = 50), psychological symptom scores were inversely correlated with PedsQL total scores, with Spearman’s coefficients ranging from −0.568 to −0.720 (all p < 0.001). Negative correlations were also observed across the individual PedsQL domains. These exploratory bivariate associations are presented in **Table 4**.

**Table 4:** Spearman correlations between psychological symptom burden and PedsQL outcomes.

Psychological measure	Physical	Emotional	Social	School	Psychosocial	Total
SCARED	-0.487***	-0.436**	-0.351*	-0.605***	-0.541***	-0.568***
RCADS-CV anxiety	-0.654***	-0.517***	-0.502***	-0.613***	-0.644***	-0.677***
RCADS-CV depression	-0.496***	-0.573***	-0.577***	-0.576***	-0.676***	-0.656***
RCADS-CV total	-0.670***	-0.566***	-0.548***	-0.637***	-0.689***	-0.720***
CDI	-0.365**	-0.635***	-0.428**	-0.484***	-0.641***	-0.593***

Values are Spearman’s rank correlation coefficients (r_s_ ). All correlations were calculated in the combined CPP–IPT sample (n = 50). *p<0.05; **p<0.01; ***p<0.001.

These exploratory bivariate analyses were not adjusted for covariates or multiple testing. Significance markers represent unadjusted two-sided p values. Physical, emotional, social, and school refer to the corresponding PedsQL functioning domains; psychosocial and total refer to the Psychosocial Health Summary Score and Total Scale Score, respectively. SCARED, Screen for Child Anxiety Related Emotional Disorders; RCADS-CV, Revised Child Anxiety and Depression Scale–Child Version; CDI, Children’s Depression Inventory; PedsQL, Pediatric Quality of Life Inventory.

### Exploratory hierarchical regression analysis

In Step 1, endocrine phenotype, BMI SDS, and duration of breast-development symptoms accounted for 2.8% of the variance in PedsQL total scores (R² = 0.028, adjusted R² = −0.036; F[3,46] = 0.437, p = 0.728).

After SCARED and CDI total scores were added in Step 2, the model accounted for 52.5% of the variance (R² = 0.525, adjusted R² = 0.472; F[5,44] = 9.745, p < 0.001). The increase in R² was 0.498 (F-change[2,44] = 23.079, p < 0.001).

In the final model, higher SCARED and CDI scores were associated with lower PedsQL total scores after adjustment for the other included variables: SCARED, B = −0.577, β = −0.382, 95% CI for B −0.950 to −0.204, p = 0.003; CDI, B = −1.483, β = −0.481, 95% CI for B −2.261 to −0.705, p < 0.001. No statistically significant adjusted associations were detected for endocrine phenotype (p = 0.779), BMI SDS (p = 0.874), or symptom duration (p = 0.892).

Variance inflation factors ranged from 1.046 to 1.453, and the maximum Cook’s distance was 0.222.

## Discussion

The present study directly compared anxiety and depressive symptoms and multidimensional health-related quality of life (HRQoL) in girls with central precocious puberty (CPP) and isolated premature thelarche (IPT). No statistically significant between-group differences were detected after false-discovery-rate correction. In the combined sample, higher anxiety and depressive symptom scores were associated with lower HRQoL. These findings describe associations within a clinical sample and do not establish the relative importance of endocrine and psychological factors. Importantly, the absence of statistically significant between-group differences should not be interpreted as evidence of psychological equivalence between CPP and IPT.

Our findings are particularly relevant to the limited literature directly comparing CPP and premature thelarche. Yılmazer et al. (11) found no statistically significant overall differences in physical, psychosocial, or total PedsQL scores among children with CPP, premature thelarche, and healthy controls. In contrast, Donbaloglu and Bostan (12) reported higher RCADS scores and lower physical, psychosocial, and total PedsQL scores among girls with premature thelarche compared with those with idiopathic CPP. These differing results indicate that a consistent phenotype-specific psychological profile has not been established. Differences in assessment timing, treatment exposure, developmental characteristics, and sampling may contribute to the variability, although the present study cannot determine which factors explain the discrepancies.

Köprülü and Yazkan Akgül (13) also reported no statistically significant differences in anxiety–depression scores or HRQoL among children with newly diagnosed CPP, children receiving treatment for more than one year, and healthy controls. Their findings provide additional evidence of heterogeneity in reported psychological outcomes. However, the cross-sectional comparison of separate treated and untreated groups, without pretreatment psychological assessments in the treated group, cannot establish treatment-related changes. This distinction is important when comparing studies conducted at diagnosis with those including children already receiving treatment.

Studies using healthy comparison groups address a different question from the present CPP–IPT comparison. Çoban et al. (4) identified greater peer victimization among girls with CPP, while Kardaş et al. (5) reported associations involving child anxiety, HRQoL, maternal anxiety, and pubertal stage. Turan and Akca (28) found lower child- and parent-reported HRQoL in children with precocious puberty than in healthy children. These observations highlight potentially relevant emotional, family, and peer experiences. However, our comparison neither measured nor controlled for these experiences. The presence of early breast development in both groups therefore should not be interpreted as equivalent psychosocial exposure, and our findings cannot separate endocrine from psychosocial contributions.

Assessment timing is particularly relevant to interpreting our results. The assessment ages of approximately 8.3 years were distinct from the parent-reported ages at first recognition of breast development, which preceded 8 years in both groups. Psychological questionnaires were administered on the same day as the endocrinological diagnostic evaluation in both groups, and all assessments in the CPP group preceded GnRH agonist treatment. This approach avoided comparing pretreatment CPP assessments with post-treatment assessments. Nevertheless, a common assessment schedule does not eliminate differences in pubertal progression, family concern, or experiences preceding referral.

Longitudinal evidence also cautions against interpreting a cross-sectional finding as reassurance about subsequent psychiatric risk. Dinkelbach et al. (8) and Hueg et al. (9) reported elevated risks of several psychiatric disorders among individuals with CPP compared with population referents. These studies evaluated clinically recorded disorders over extended follow-up, whereas our study examined questionnaire scores at one assessment. Their findings are therefore not directly contradicted by the absence of statistically significant CPP–IPT differences in our sample. Conversely, our results do not demonstrate that the two groups will follow similar psychological trajectories.

The inverse associations between psychological symptom scores and HRQoL require particular caution. SCARED, RCADS-CV, CDI, and PedsQL were all child self-report measures, creating the possibility of common-method variance. There is also conceptual overlap between internalizing symptoms and aspects of HRQoL, particularly emotional functioning. Accordingly, the observed correlations are not evidence of a novel or independent psychological mechanism. Using complementary symptom questionnaires helps describe consistency across instruments but does not provide independent confirmation when the measures share respondents and content. Psychological distress may contribute to poorer perceived functioning, impaired functioning may intensify emotional symptoms, or both may arise from shared psychosocial vulnerabilities. The clinical contribution is therefore the description of emotional symptoms alongside functional well-being in these two clinical groups, rather than the discovery that psychological distress and HRQoL are related.

In the exploratory regression, SCARED and CDI scores remained associated with PedsQL total scores after adjustment for endocrine phenotype, BMI SDS, and symptom duration. The regression model included five predictors in a sample of only 50 participants and should therefore be regarded as exploratory rather than confirmatory. Adjustment for these variables does not remove conceptual overlap or unmeasured confounding, and the findings should be interpreted as adjusted associations rather than evidence of causality. Moreover, the nonsignificant coefficient for endocrine phenotype cannot establish that hypothalamic–pituitary–gonadal axis activation has no psychological relevance. Although FDR correction was applied to the specified between-group outcome families, correlation and regression analyses remained exploratory and were not adjusted for multiple testing.

From a clinical perspective, elevated screening scores in both groups suggest that emotional and functional concerns should be considered when evaluating girls with early breast development. Importantly, the SCARED and CDI thresholds used in our study represent screening cutoffs and should not be interpreted as psychiatric diagnoses. Clinical assessment should consider the persistence of symptoms and their impact on daily functioning rather than questionnaire scores alone. The present findings do not establish the benefit of routine psychiatric referral for every child, nor do they demonstrate that psychological intervention would improve HRQoL. These questions require studies designed to evaluate clinical assessment and intervention strategies.

Several limitations should also be acknowledged. The single-center sample was small and the diagnostic groups were unequal in size, limiting precision and the ability to detect modest differences. The absence of a healthy-control group means that we cannot determine whether either clinical phenotype has greater psychological symptom burden than girls without early breast development. The restricted age range limits generalizability to younger children and later developmental stages. Parent-reported first recognition of breast development is susceptible to recall error and may not precisely identify biological onset. Exclusion of participants with established psychiatric disorders or psychotropic medication use may have restricted the observed range of psychiatric morbidity. Body image, peer victimization, parental anxiety, and other potentially relevant psychosocial factors were not directly assessed. RCADS-CV scores were analyzed as raw dimensional scores without locally validated normative T-score interpretation, limiting population-referenced conclusions. No anxiety or depression measure was designated *a priori* as the primary outcome. These limitations should be weighed against the direct clinical comparison and the consistent timing of assessments across groups.

Future studies should examine change over time rather than infer trajectories from a single assessment. Deardorff et al. (29) and Joinson et al. (30) provide broader longitudinal evidence that relationships between pubertal development and emotional symptoms vary with developmental and population context, although neither study specifically establishes CPP–IPT differences. Larger longitudinal studies incorporating healthy comparators, repeated endocrine and psychological assessments, multiple informants, and measures of family and peer experiences would help clarify these relationships and their clinical implications.

## Conclusion

In girls presenting with early breast development, no statistically significant differences in anxiety symptoms, depressive symptoms, or health-related quality of life (HRQoL) were detected between CPP and IPT after correction for multiple comparisons. In the combined sample, higher anxiety and depressive symptom scores were associated with poorer HRQoL. These associations require cautious interpretation given the small sample size, cross-sectional design, and conceptual overlap between psychological symptom measures and HRQoL. The absence of statistically significant group differences does not establish psychological equivalence or exclude a contribution of HPG-axis activation to psychological well-being. Larger longitudinal studies with healthy comparison groups are needed to confirm these findings and clarify psychological trajectories in girls with CPP and IPT.

## Data Availability

The datasets generated and/or analyzed during the current study are not publicly available due to ethical and confidentiality restrictions but are available from the corresponding author upon reasonable request and with appropriate institutional approval.
